# A Novel Role for Connexin Hemichannel in Oxidative Stress and Smoking-Induced Cell Injury

**DOI:** 10.1371/journal.pone.0000712

**Published:** 2007-08-08

**Authors:** Srinivasan Ramachandran, Lai-Hua Xie, Scott A. John, Shankar Subramaniam, Ratnesh Lal

**Affiliations:** 1 Center for Nanomedicine, The University of Chicago, Illinois, United States of America; 2 Cardiovascular Research Laboratory, University of California at Los Angeles, California, United States of America; 3 Department of Bioengineering, University of California at San Diego, La Jolla, California, United States of America; Universität Heidelberg, Germany

## Abstract

Oxidative stress is linked to many pathological conditions, including ischemia, atherosclerosis and neurodegenerative disorders. The molecular mechanisms of oxidative stress induced pathophysiology and cell death are currently poorly understood. Our present work demonstrates that oxidative stress induced by reactive oxygen species and cigarette smoke extract depolarize the cell membrane and open connexin hemichannels. Under oxidative stress, connexin expression and connexin silencing resulted in increased and reduced cell deaths, respectively. Morphological and live/dead assays indicate that cell death is likely through apoptosis. Our studies provide new insights into the mechanistic role of hemichannels in oxidative stress induced cell injury.

## Introduction

Oxidative stress is associated with senescence, neurodegenerative disorders such as Alzheimer's and Parkinson's diseases, and acute traumatic and ischemic insults [1], [2], [3]. Uncompensated oxidative stress, as would occur under sustained harmful particulate or pollutant exposures, results in cellular toxicity by activating apoptotic or necrotic pathways depending on the degree of imbalance and the metabolic state of the cell [4], [5]. Cigarette smoke exposure, a common health issue, has been implicated in oxidative stress and the concomitant cell apoptosis [6], [7].

The cellular mechanisms of apoptosis induced by smoking, in particular and oxidative stress, in general are complex and poorly understood. Extracellular oxidative stress is reported to induce cell death through a variety of mechanisms including plasma membrane receptors, non-selective channels, and or endocytosis [8]. Both intercellular and extracellular communications could play important roles in activating cell signal transduction pathways underlying these apoptotic mechanisms.

Gap junctional communication between adjacent cells is the most direct intercellular communication pathway that is implicated in apoptosis [9]. Each gap junction communication channel is made of two hemichannels (commonly called connexons), one each from the apposing cells. Each hemichannel is made of six connexin (Cx) proteins that are assembled in the intracellular compartment and transported to the cell membrane [10], [11], [12], [13]. Hemichannels are also shown to be present in the non-junctional portions of the plasma membrane [14], [15], [16], [17]. These hemichannels have been shown to form aqueous conduits between the inside and outside of the cell allowing the passage of molecules up to ∼1000 Daltons (Da) [15], [18], [19], [20], [21]. The opening of theses hemichannels, either in response to physiological perturbations, such as a low extracellular calcium [14], [15], [22], [23] or pathological perturbations, such as ischemia [24], [25] would allow the movement of molecules down their respective concentration and electrochemical gradients. Such molecular transport can lead to a loss of ionic homeostasis and destabilization of the membrane potential, which could further influence hemichannel activity. In addition, given the size of the biologically relevant oxidative stress inducing molecules (<1000 Da) [26], an open hemichannel would also allow the direct uptake of perturbants themselves. Thus, in response to external pathological insults, hemichannels can alter cell physiology directly [27].

We demonstrate here a novel role for hemichannels: an oxidative stress induced cell death mediated directly through open hemichannels. In examining the role of hemichannel activity, our studies show that both cigarette smoke extract (CSE) and its prominent reactive oxygen species (ROS) component, H_2_O_2_ (for review see [28]) cause membrane depolarization and open hemichannels. The open hemichannels facilitate the entry of these molecules and that in turn causes cell injury and death.

## Results

### Oxidative stress (CSE/H_2_O_2_) induces hemichannel opening

The primary target sites of cigarette smoking are the lungs and heart. We wanted to examine the role of hemichannels in smoking and oxidative stress induced injury in these tissues. Cx43 is the predominant connexin expressed in the heart; Cx32 and Cx43 are the predominant connexins expressed in the lungs. For our study, we chose L2 cells which expresses both Cx32 and Cx43; Marshall cells (MC) which expresses only Cx43; and Cx-deficient N2A cells to serve as control. Cx-expression in the non-junctional (hemichannel) plasma membrane portions were evaluated by immunofluorescent labeling using anti-Cx antibodies. As shown in the supplement section (Fig. S1), both MC and L2 cells express high level of connexins while N2A cells have none-to-little Cx-labeling.

**Figure 1:**
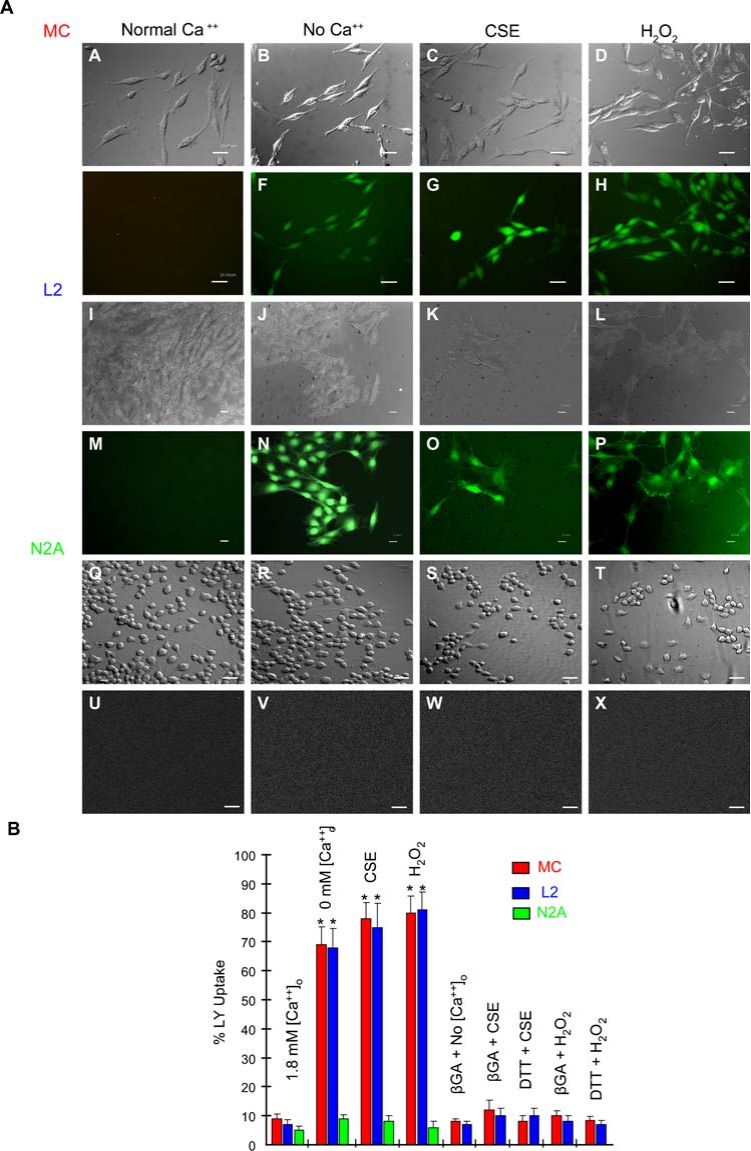
Connexin hemichannels open under oxidative stress independent of calcium gating mechanism. (A). In the normal Ca^++^ containing extracellular medium, no significant LY uptake was observed for MC (A,E) and L2 (I,M) cells. In Ca^++^ free medium, both cell types show LY uptake (B,F; J,N) consistent with hemichannel behavior. In the presence of normal Ca^++^ medium, both cell lines allow LY uptake under oxidative stress induced by 10% CSE (C,G; K,O) and 1 mM H_2_O_2_ (D,H; L,P). Hemichannel mediated dye uptake was not observed in Cx-deficient N2A cells from Ca^++ ^free medium (R,V) as well as under oxidative stress conditions (S,W; T,X). Scale bar: 20 µm. (B). Histogram representation of the LY uptake studies in the cell lines examined under different treatment conditions. Comparison of different treatment groups made against the control (*p<0.0001), values expressed as mean ± SE.

The functional state of hemichannels was assessed by the cellular uptake of Lucifer yellow (LY) from a Ca^++^ free extracellular medium [14], [15]. Figure **1A**, shows an example of LY uptake in MC as depicted in panels (B,F) and L2 cells (J,N) but not in N2A cells (R,V). On the other hand, no significant LY uptake was observed in the presence of normal Ca^++ ^[1.8 mM] in the medium ((A,E), (I,M) and (Q,U)). These results indicate hemichannels open in Ca^++^ deficient medium under non-stress conditions.

Hemichannel activity under oxidative stress (induced by CSE and H_2_O_2_) was examined by LY uptake from the extracellular medium containing normal Ca^++^. The results show that, oxidative stress opened hemichannels in MC and L2, but not in N2A cells, i.e., the oxidative stress induced hemichannel opening is non-calcium dependent. Cx-expressing cells pretreated with hemichannel blockers, namely 18-β-glycyrrhetinic acid (βGA) or Meclofenamic acid (MFA) did not show significant LY uptake (**Fig. 1B**). These results indicate that oxidative stress induced LY uptake occurs through hemichannels. To establish the role of oxidative stress in hemichannel opening, we used an antioxidant, dithiothreitol (DTT) to abolish the induced oxidative stress. Cells pretreated with DTT did not show significant dye uptake under oxidative stress (**Fig.** **1B**) thus suggesting oxidative stress is the underlying cause of hemichannel opening.

The threshold concentration of CSE and H_2_O_2_ required to open hemichannels was determined by LY uptake at different concentrations of CSE and H_2_O_2_. The results show a clear dose-dependent LY uptake for concentrations as low as 1% CSE and 50 µM H_2_O_2_ (Fig. S2). For further study however, 10% CSE and 1mM H_2_O_2_ were used. This selection was based on our preliminary observations that these oxidative stress levels resulted in significant cellular changes within a short time frame of 6 hrs. This short time frame avoids issues of cell viability under *in vitro* imaging conditions. If lower concentrations were used, up to 24 hrs of cell incubation was required to obtain similar cellular changes (data not shown) and the normal cell viability was uncertain.

To rule out the possibility of non-hemichannel mediated mechanisms such as purinergic pathways, TRP channels and endocytosis in the dye uptake under oxidative stress conditions, series of experiments were performed under identical conditions. To exclude purinergic receptor mediated pathway, a double dye uptake study was carried out in two steps. In the first step, oxidative stress was induced in Cx-expressing MC along with Ethidium Bromide (EtBr) for 10 min and washed with a normal Ca^++ ^containing buffer. In the second step, they were treated with a purinergic blocker (PPADS) for 10 min and further subjected to oxidative stress along with LY (Fig. S3). All the cells which showed EtBr uptake in the first step (where both hemichannels and putative purinergic receptor mediated pathways could have been open) also showed LY uptake in the second step (where the purinergic receptors were blocked by its inhibitor) confirming that dye transfer was not through purinergic receptor mediated pathways.

TRP channels are ubiquitously distributed in most mammalian cells [29], [30] and purinergic receptors are widely distributed in the nervous system and many other tissues [31]. Thus, it is reasonable to assume that they are also expressed in N2A cells. Demonstrating the lack of dye uptake in Cx-deficient N2A cells under oxidative stress or upon removal of extracellular Ca^++^ in the presence of purinergic blocker would suggest that TRP channels are not contributing to dye uptake. This was confirmed by LY uptake experiment in N2A cells in the presence of purinergic blocker (Fig. S4). No dye uptake was observed under these conditions.

### Oxidative stress (CSE/H_2_O_2_) opens hemichannel by depolarizing the membrane potential

**Figure 2:**
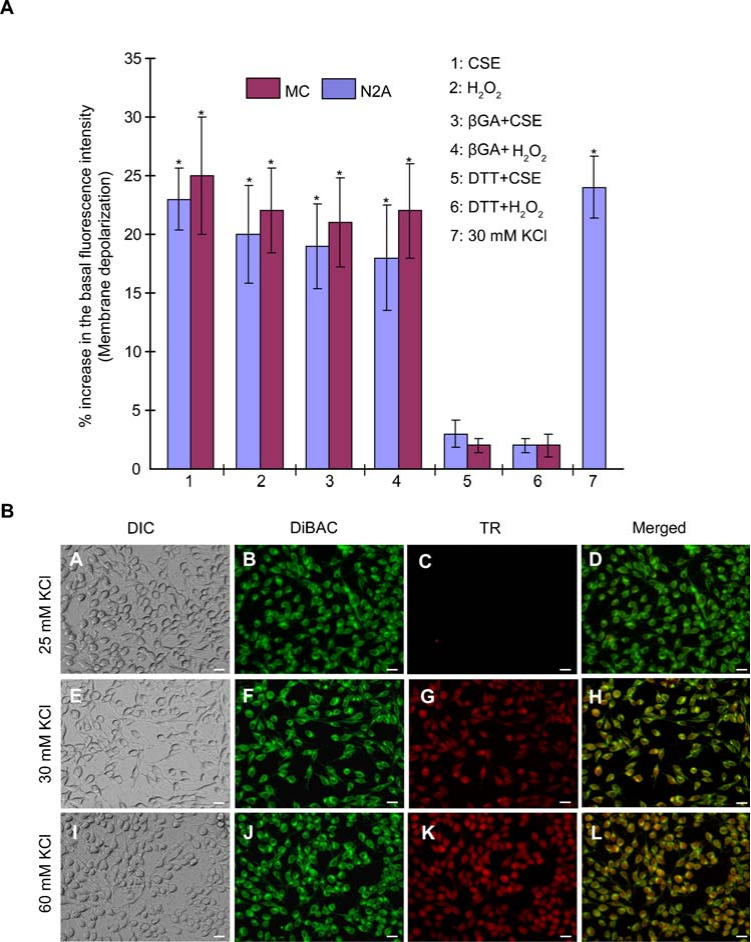
Oxidative stress induces membrane depolarization and opens hemichannel. (A). Plasma membrane depolarization induced by oxidative stress (H_2_O_2_/CSE) was detected by voltage sensitive dye (DiBAC_4_(3)). The histogram summarizes the results of change in the fluorescence intensity (indicative of membrane depolarization) as percentage of their basal fluorescence value (*p<0.0001). Error bar indicates SE of the mean. Both, Cx-expressing (MC) and Cx-deficient (N2A) cells show increase in their basal fluorescence (∼20–25%) after 3 min treatment with 1 mM H_2_O_2_ and 10% CSE with and without βGA pretreatment, while there was none-to-little increase in fluorescence (<3%) in DTT pretreated cells. (B). Quantitative estimation of the level of membrane depolarization required to open hemichannels. Membrane depolarization and hemichannel opening were assayed simultaneously using voltage sensitive dye (green) and hemichannel permeable TR. Hemichannels opened at 30 mM external K^+^ (E–H) and above (I–L). No dye uptake was observed at or below 25 mM K^+^ (A–D). Hemichannel opening at 30 mM K^+^ concentration corresponds to −38 mV of membrane voltage as calculated using the Nernst equation. Scale bar: 20 µm.

Earlier studies have reported that oxidative stress can induce cell membrane depolarization [32]. We examined this possibility using the voltage sensitive dye DiBAC_4_(3). Cells treated with CSE or H_2_O_2_ showed significant increase (∼25%) in voltage sensitive fluorescence level consistent with the membrane depolarization (**Fig.** **2A**). Cells pretreated with DTT showed inhibition of oxidant-induced fluorescence (<3%) indicating none-to-little change in the membrane potential. We then examined whether the oxidative stress induced membrane depolarization is dependent on connexin expression/activity. Significantly, both Cx-deficient N2A cell as well as hemichannel blocked (βGA) MC showed significant membrane depolarization under oxidative stress.

**Figure 3:**
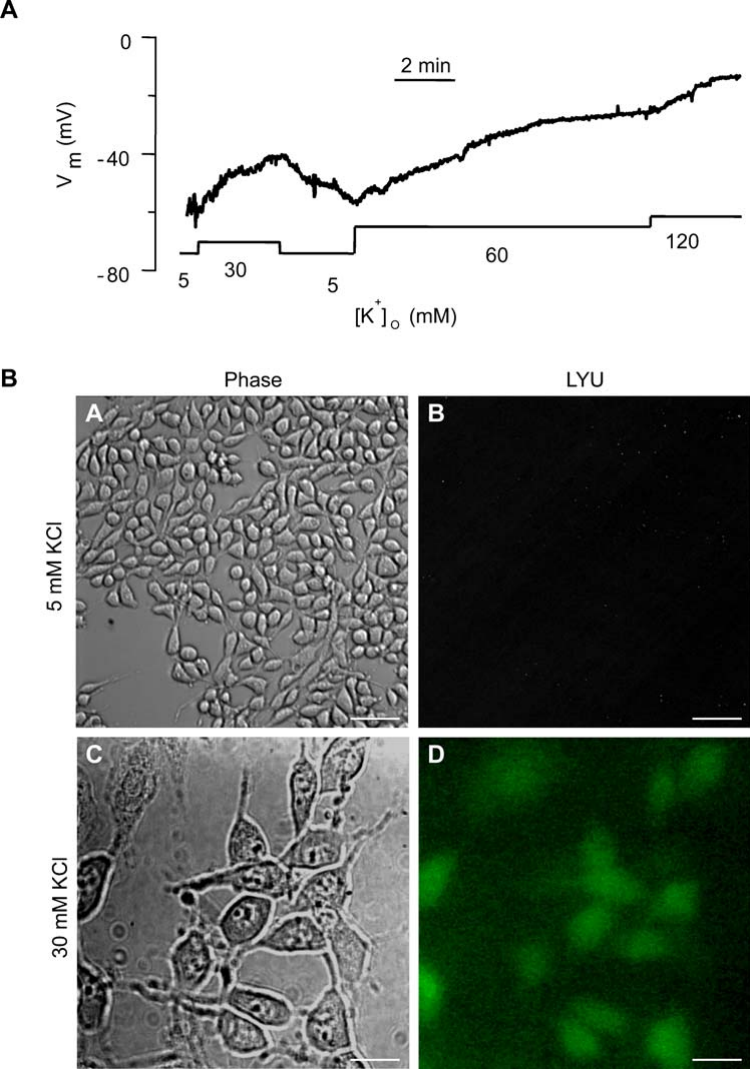
Electrophysiological measurement of the membrane potential and dye uptake in MC. (A). The membrane potential trace was measured using the whole-cell patch clamp technique from a cultured MC. Changing of [K^+^]_o_ to various values is indicated by the bars under the trace. The pseudo-steady-state membrane potential values were −61, −42, −21 and −12 mV at [K^+^]_o_ of 5, 30, 60 and 120 mM, respectively. The intracellular K^+^ concentration was ∼140 mM. (B). Uptake of LY dye in the extracellular solution of 30 mM KCl (C,D). The cells were incubated for 10 min before being washed with a LY dye-free buffer solution containing 5 mM KCl solution for 4–5 min. For comparison, dye uptake at 5 mM KCl is included (A,B). Scale bars: 25 µm in A,B and 10 µm in C,D.

The abovementioned findings, namely the oxidative stress induced hemichannel opening (**Fig.** **1**) and membrane depolarization (**Fig.** **2A**) suggest that the membrane depolarization is the underlying mechanism for hemichannel opening. To examine the possibility that membrane depolarization, irrespective of its underlying mechanism, could open hemichannels, we depolarized MC by increasing the external K^+^ and observed uptake of Texas Red (TR, 625 Da) dye which is hemichannel permeable [33]. We then estimated the level of membrane depolarization required to open hemichannel. Defined amounts of K^+^ concentrations were applied externally to MC that were pre-loaded with DiBAC_4_(3) and valinomycin along with TR. Results indicate that hemichannels open (**Fig.** **2B**, G) at the membrane potential of ∼−38 mV, as computed using the Nernst equation for 30 mM extracellular K^+^ (assuming 140 mM K^+^ inside the cell). This estimation was further verified by direct electrophysiological measurements on MC using the whole-cell patch clamp technique (see methods). With 5 mM extracellular K^+^, MC has a resting membrane potential of −61 mV. Switching the extracellular K^+^ concentration to 30, 60 and 120 mM sequentially resulted in measured membrane potential values of −42, −21 and −12 mV, respectively (**Fig.** **3A**). Significantly, detectable level of LY dye uptake was observed at 30 mM K^+^ concentration while no dye uptake was seen at 5 mM K^+^ (**Fig.** **3B**).

### Oxidative stress induces hemichannel dependent cell death

**Figure 4:**
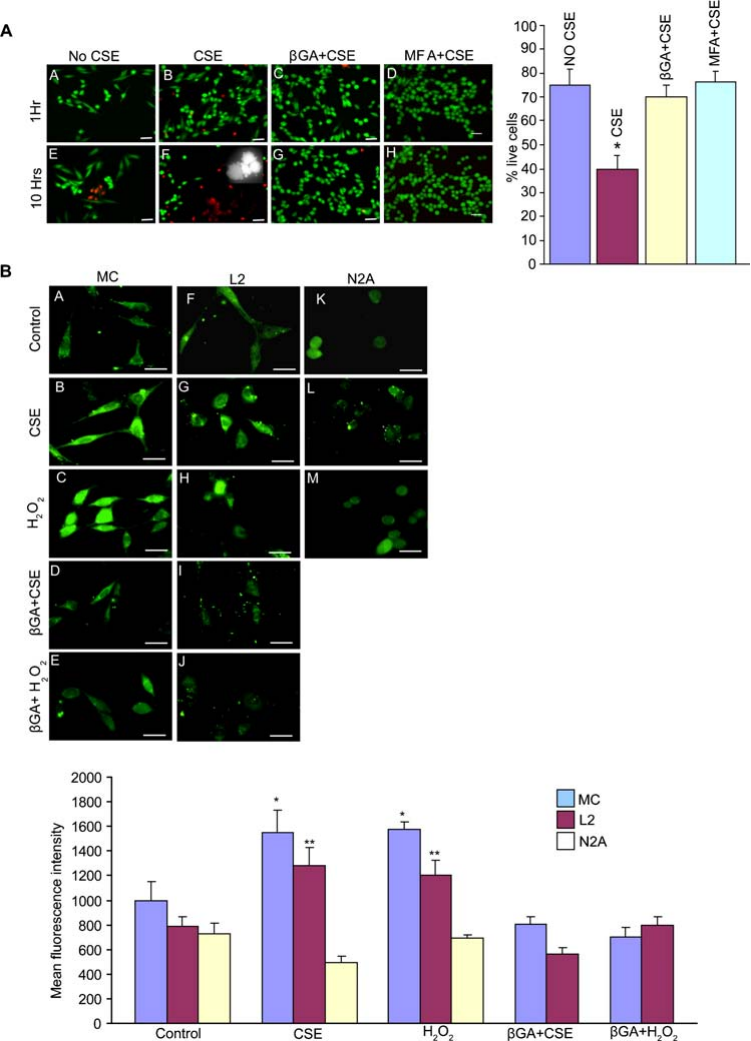
CSE cause cell death and intracellular oxidative stress. (A). Live/dead cell assay to estimate hemichannel mediated cell death under oxidative stress. Early cell death was observed in CSE treated cells (red fluorescent cells; EtBr homodimer dye) at the end of 10 hrs (F) compared to control (E), βGA pretreated cells (G) and MFA pretreated cells (H), where more live cells (green fluorescent cells; Calcein AM dye) are seen. Cell death in F was predominantly apoptotic as evident from the morphology of the dying cells viz., cell shrinkage, fragmentation into membrane bound apoptotic bodies etc., (inset in panel F). The histogram shows the % of live cells at the end of 10 hrs in all four categories. Error bar indicates SE of the mean (*p<0.012). Scale bar: 20 µm. (B). Oxidative stress molecules enter into the cells through open hemichannels as detected by ROS sensitive dye (Carboxy-H_2_DCFDA). Cx-expressing cells (MC and L2), showed increase in fluorescence after 10 min treatment with CSE (B,G) and H_2_O_2 _(C, H) compared against their corresponding controls (A,F). Similarly, βGA pretreatment decreased such change with CSE (D,I) as well as with H_2_O_2_ (E,J). Cx-deficient N2A cells showed no significant change from their control (K) under same treatment conditions (L,M). Scale bar: 20 µm. Histogram summarizes the mean fluorescence intensities of different treatment conditions of all three cell lines subjected to oxidative stress. Error bar indicates SE of the mean (*p<0.0007, **p<0.0015).

Oxidative stress induced cell injury was measured by live/dead assay on MC incubated with CSE with and without hemichannel blockers (βGA/MFA). The results indicate that at the end of 10 hrs, 40% of cells were alive in the CSE treated group, 70% in βGA+CSE group, 76% in MFA+CSE group and 75% in control group. Cell morphology assay revealed a strong likelihood of “massive” apoptosis (cell shrinkage, and fragmentation into membrane bound apoptotic bodies) in the CSE treated cells (**Fig.** **4A**, F inset) as opposed to βGA/MFA/control groups. These results show that oxidative stress opens hemichannels that probably trigger cell death by apoptotic mechanisms while inhibition of hemichannels delays/prevents cell death.

### CSE and H_2_O_2_ enter through the open hemichannels and induce cell death

We examined whether CSE and H_2_O_2_ could induce cell death directly by entering through open hemichannels. We measured CSE and H_2_O_2_ cellular uptake using a ROS sensitive dye (carboxy-H_2_DCFDA), cells preloaded with ROS sensitive dye when subjected to oxidative stress displayed an increase in ROS specific fluorescence in Cx-expressing cells, while no such change was observed in the N2A cells (**Fig.** **4B**). Pre-treatment with a hemichannel blocker (βGA) prevented increase in ROS specific fluorescence (**Fig.** **4B**) thus suggesting the cellular entry of CSE/H_2_O_2 _was facilitated, in part, through open hemichannels.

### Cx43 expression in Cx-deficient cell facilitates oxidative stress induced cell death

**Figure 5:**
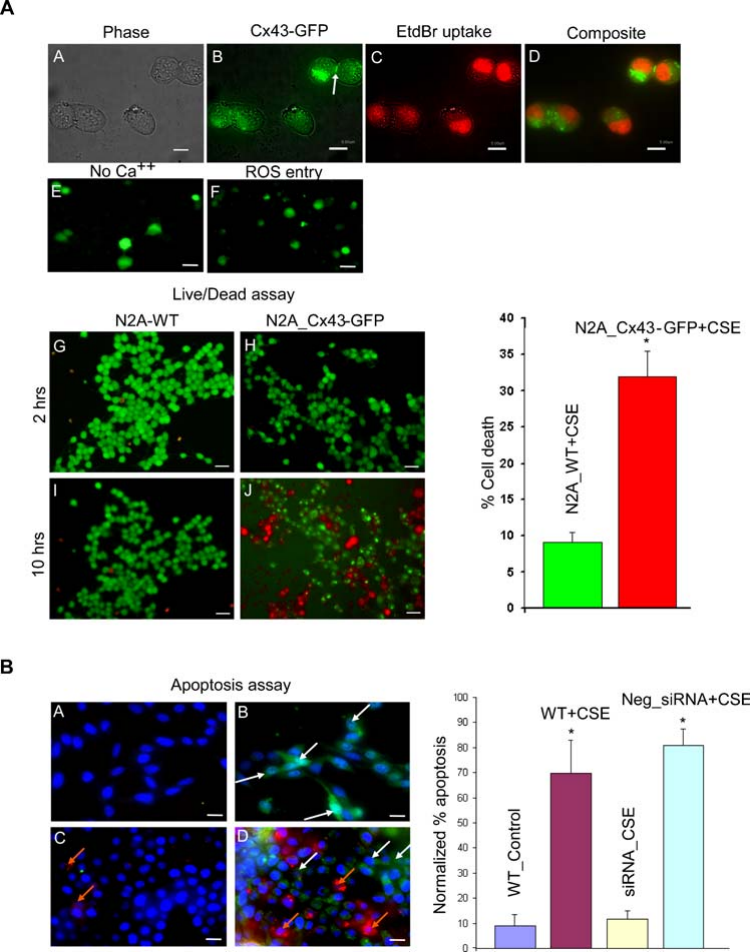
Hemichannel mediates cell death under oxidative stress as evidenced by gene transfection and silencing techniques. (A). Cx43-GFP transfected N2A cells produced hemichannels and gap junctions (B, white arrow indicating typical distribution of Cx-channels in the cell-cell contact regions) and they responded to low Ca^++ ^medium (E, LY uptake) and oxidative stress (C, EtBr uptake induced by CSE). The open hemichannels permit direct entry of ROS into the cells as detected by ROS sensitive dye (F). Live/dead assay showed significant cell death induced by CSE in Cx-transfected N2A cells (33%) (H,J) compared against their wild type N2A cells (9%) (G,I) (*p<0.0072). Scale bars (A–D): 5 µm, (E–J): 20 µm. (B). Apoptosis assay on Cx43 silenced MC after 10 hrs of induced oxidative stress (10% CSE). (A) Wild type MC in normal media (control) show live cells (blue nuclei, Hoechst 33342 stained). (B) Wild type MC in CSE show apoptotic cells (green cells, YO-PRO stained) indicated by white arrows. (C) Cx43 silenced MC show live cells (blue nuclei) and few necrotic cells (Red nuclei, Propidium Iodide) indicated by orange arrows. (D) Negative-siRNA transfected MC shows more apoptotic and necrotic cells than the wild type MC. Histogram shows the % apoptosis observed in different treatment groups (*p<0.0001). Scale bar: 5 µm

To further confirm the direct involvement of hemichannels in oxidative stress induced cell death, we transfected Cx43-Green Fluorescent Protein (Cx43-GFP) plasmids into wild type N2A cells and carried out a series of experiments. The presence of connexin channels and their activity under oxidative stress were assessed by fluorescence microscopy and dye uptake (EtBr) studies, respectively. To exclude any role of purinergic pathways in dye uptake, we used purinergic blocker PPADS. Results are summarized in **Fig.** **5A**. Panel A shows a phase image of cells in the field, panel B shows Cx43-GFP distribution and panel C shows EtBr uptake. Panel D is the composite image (of B and C) indicating the presence of functional hemichannels. We further show that these expressed hemichannels uptake LY in the Ca^++ ^free medium (E) and allow direct uptake of ROS agents through open hemichannels, an increase in the ROS specific intracellular fluorescence (F).

Live/dead cell assays indicate that upon CSE exposure, there is an increase in cell death after 10 hrs in Cx-transfected cells (33%) (H, J) compared to the wild type N2A cells (9%) (G, I). Taken together these data strongly suggests that (a) Cx43-GFP transfected N2A cells form functional hemichannels, (b) they open in response to low extracellular calcium and oxidative stress, and (c) they facilitate rapid entry of ROS agents through open hemichannels and mediate cell death.

### Silencing Cx43 in Marshall Cell improves their survival under oxidative stress

Post-transcriptional gene silencing of Cx43 was achieved in MC by short interfering segments of RNA (siRNA) targeted against the intracellular loop of the Cx43 [34] . Down regulation of Cx43 was observed by lower incidence of LY uptake in a Ca^++^ free media (data not shown). An apoptosis specific assay was carried out by fluorescence microscopy (Hoechst 33342, YO-PRO and Propidium Iodide staining for live, apoptotic and necrotic cells, respectively) (**Fig.** **5B**). In response to CSE, apoptosis in the wild type MC increased (70%) (B) compared against their control group (9%) (A). Silencing Cx43 prevented apoptosis (12%) (C) and improved cell survival when compared against the negative control siRNA (81%) (D) and wild type MC (70%), under same conditions.

## Discussion

Many native cells and cell lines express connexin proteins. These connexin proteins form functional hemichannels and gap junctions. In this study, we show that oxidative stress induces hemichannel opening that facilitates cell death. We also demonstrate by imaging and electrophysiological measurements that the mechanism of hemichannel opening implicates direct depolarization of the plasma membrane due to oxidative stress. Open hemichannels promote toxicity in two ways - by facilitating the direct entry of toxic molecules (CSE/H_2_O_2_) as we have demonstrated and through the loss of vital metabolites such as ATP and NAD [35], [36], [37], [38], [39].

It has been shown previously that the opening of hemichannels is influenced by Ca^++ ^dependent gating [14], [15], [22], [23]. However, in the presence of normal extracellular Ca^++^, our experiments show that CSE and H_2_O_2_ open hemichannels suggesting this opening is independent of the Ca^++ ^dependent gating mechanisms. The primary suggestion for the opening of hemichannels came from dye uptake experiments and from the inhibition of dye uptake by hemichannel blockers βGA and MFA. However, both βGA and MFA have not been conclusively shown to be specific to hemichannels alone. On the other hand, the lack of dye uptake in Cx-deficient N2A cells, dye uptake in Cx-transfected N2A cells and reduced uptake in Cx-silenced MC under the same experimental conditions directly implicates connexin hemichannels in the dye uptake. Involvement of non-hemichannel transport mechanisms such as purinergic receptor mediated pathways, TRP channels and other nonspecific mechanisms like endocytosis is also a possibility.

Non-specific dye transfer due to endocytosis and other membrane opening mechanisms are temperature dependent [40]; dye uptake experiments carried out at 4°C show no perceptible difference in dye uptake validating the absence of these non-specific mechanisms (data not shown). Purinergic receptor pathways were excluded by using broad spectrum purinergic blockers in a double dye study in the Cx-expressing cells (Fig. S3). Also, the Cx43-GFP transfected N2A cells showed dye uptake under oxidative stress in the presence of purinergic blocker (**Fig.** **5A, A**–D) thus confirming the exclusion of purinergic mediated pathways in the dye transfer.

However, we could not rule out the involvement of TRP channels directly and conclusively in the dye transfer because (a) currently there is no specific TRP channels blocker is available and (b) all the TRP channel blockers available also block the hemichannels. In addition, demonstration of the lack of dye uptake in N2A cells under oxidative stress in the presence of purinergic blocker allows us to reasonably conclude that though TRP channels are ubiquitously expressed they do not play a role in dye uptake under our experimental conditions. As a further validation of this reasoning, Cx43-GFP transfected N2A cells showed dye uptake under identical treatments. Thus the only viable mechanism for the uptake of molecules appears to be transport through connexin hemichannels.

The mechanism of hemichannel opening under oxidative stress has not been studied previously. Earlier studies have shown that oxidative stress induces membrane depolarization [32] and therefore we examined whether hemichannels open due to the membrane depolarization. Data (**Fig.** **2A**) demonstrate that (a) oxidative stress induces membrane depolarization in both Cx-expressing (MC) and Cx-deficient (N2A) cells, and (b) is not affected by hemichannel inhibitors but is affected by DTT. Importantly, both βGA and DTT prevent dye uptake i.e. prevent hemichannels from opening under oxidative stress (**Fig.** **1B**). This leads to two important conclusions; (a) oxidative stress induced membrane depolarization is independent of Cx expression or its functional activity and (b) βGA prevents dye transfer by blocking the hemichannel while DTT abolishes membrane depolarization thus preventing hemichannel opening. This data confirms that membrane depolarization is the underlying mechanism of oxidative stress induced hemichannel opening. Since hemichannels are large (10–15 Å) nonspecific channels with high conductance (over 300 pS) [21], open hemichannels would lead to further depolarization of the cell membrane. In this study, we measured only the steady state change in the membrane fluorescence after 3 min of applying the stimulus. Studying the fluorescence change with higher time scale resolution would reveal the depolarization events secondary to hemichannel opening.

The double dye experiment results (**Fig.** **2B**) show that hemichannels open above 30 mM external K^+^ and allow dye uptake from the medium. The computed membrane potential at 30 mM K^+ ^using the Nernst equation, assuming [K^+^]_i_ as 140 mM was −38 mV. Earlier reports show hemichannels open only when the membrane is depolarized to positive potential [41], [42]. Direct electrophysiological measurement of membrane potential with 30 mM external K^+ ^was found to be −41 mV, while the physiological resting membrane potential at 5 mM external K^+^ was −64 mV. We have shown in this study that hemichannels permit transport of dyes at −38 mV indicating that at least a subset of the hemichannels are open at this potential. We note that while conductance assays require higher open probability for detection, dye permeability experiments are more sensitive and can detect even a small probability of opening.

Open hemichannels could lead to a loss of ionic homeostasis and key metabolites [35], [36], [37], [38], [39], and massive Ca^++ ^uptake which will then overload the cytosol with Ca^++^ [15] and induce Ca^++^-dependent kinases, phospholipases, proteases, ATPases, endonucleases and cause collapse of the mitochondrial membrane potential, all of which then predispose the cell to death [4], [5], [43], [44], [45], [46], [47], [48]. Results from our live/dead cell assay affirm such a possibility in the connexin expressing cells. Free radicals appear to hasten cell death by opening hemichannels and tilt the balance of cell viability, while the inhibition of hemichannels with blockers prevented the premature death of cells. More importantly, expression of Cx43-GFP in the N2A cells accelerated cell death under similar treatments compared against their untransfected N2A cells. The phenotypes of the dying cells were very characteristic of apoptosis, but not specific to apoptosis itself. Studying the detailed signal transduction mechanisms underlying this death signaling is beyond the scope of this study.

The increase in intracellular ROS specific fluorescence within 10 min from external application of CSE and H_2_O_2_ in Cx-expressing cells, absence of such increase in hemichannel inhibited cells and in Cx-deficient cells strongly suggests that hemichannels facilitate direct entry of these agents and cause cell injury. This explains the early appearance of apoptotic changes in cells treated with CSE (compared against cells protected with βGA/MFA; or Cx-deficient cells) (**Fig.** **4A**, and **5B**).

Post transcriptional gene silencing of Cx43 by specific siRNA sequence results in effective down-regulation of Cx43 (∼60%). This down-regulation prevents/protects cell injury under oxidative stress. In contrast, expression of Cx43 in N2A cell accelerates cell injury under similar conditions. Taken together, these data clearly demonstrate the role of hemichannels in cell injury and death under oxidative stress. The reason for more necrotic cells observed in the siRNA treated cells was likely due to the spontaneous death of cells after reaching confluence. For effective transfection, it warrants at least 80–90% cell confluence at the time of transfection. The cells were further maintained for 36 hrs after transfection. At this time more cells undergo spontaneous cell death which was further compounded by the toxicity of the transfection reagent (lipofectamine).

**Figure 6:**
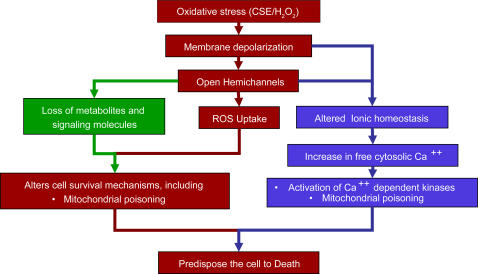
Schematic of putative mechanisms involved in oxidative stress induced hemichannel opening and cell death. Oxidative stress induced by CSE/H_2_O_2_ depolarizes the cell membrane, which opens hemichannels. Open hemichannels disturb ionic homeostasis (primarily Ca^++^, K^+^ and Na^+ ^ions) (pathway shown in purple) [15]. Increase in free intracellular calcium would activate its dependent kinases as well as load mitochondria with Ca^++ ^resulting in activation of intrinsic pathways of cell death. Open hemichannels permits transfer of apoptosis signaling molecules and metabolites including ATP [36], NAD [38], [52], etc., thus accelerating cell death (pathway shown in green). As shown in the present study, open hemichannels allow direct entry of ROS leading to cell death probably through apoptosis (pathway shown in maroon).

Our experimental observations present a likely mechanism of oxidative stress-induced hemichannel opening and apoptosis (**Fig.** **6**). This model shows that CSE/H_2_O_2_ cause plasma membrane depolarization and induce hemichannel opening. It is known that open hemichannels (a) permit loss of key cellular metabolites like ATP [36], NAD [35], [38], IP3 [39], cAMP, cGMP [37], (b) result in direct uptake of CSE/H_2_O_2_ constituents (present study), and (c) destabilize [Ca^++^]_i_ homeostasis [15] resulting in cytosolic Ca^++ ^overload, activation of Ca^++ ^dependent kinases and mitochondrial poisoning [5], [45], [46], [48], [49]. All of these events would compromise cell survival by predisposing them to apoptosis depending upon the metabolic state of the cell [44], [47]. Deciphering signal transduction pathways that ultimately leads to apoptosis was beyond the scope of this study and needs to be examined in detail. In summary, the above data suggest that hemichannels facilitate oxidative stress induced cell death, while the inhibition of hemichannels increases cell survival.

## Materials and Methods

### Reagents

Dulbecco's Modified Eagles Media (DMEM), OptiMEM I reduced serum media, and calcium free OptiMEM I and Lipofectamine 2000 were acquired from GIBCO Invitrogen. Kaighn's Modified Hams F-12 Medium (F-12K) and fetal calf serum were obtained from American Type Culture Collection. Lucifer yellow-CH (457 Da), Texas Red Sulfonyl chloride, (625 Da), Texas Red-Dextran (10,000 Da), Ethidium Bromide (394 Da) Apoptosis assay kit (Vybrant apoptosis assay kit 7), Live/Dead assay kit, voltage sensitive dye DiBAC_4_(3), Image-iT Live Green ROS detection kit and Valinomycin were purchased from Molecular probes (Eugene, OR). 18β- Glycyrrhetinic Acid (βGA), Meclofenamic acid (MFA), Pyridoxal-5′-phosphate-6-azophenyl-2′,5′-disulphonic acid sodium salt (PPADS), Oxidized-ATP (O-ATP), and H_2_O_2_ were purchased from Sigma. Anti-connexin-antibody pack containing Cx26, Cx32 and Cx43; and FITC-Goat anti-Mouse IgG were obtained from Zymed (San Francisco, CA). Chemically synthesized siRNA targeted against the intracytoplasmic loop of Cx43 (5′-GAAGTTCAAGTACGGGATT-3′) [34], HiPerfect transfection reagent, nonsense sequence with no known homology to mammalian genes (AATTCTCCGAACGTGTCACGT) with rhodamine modification at the 3′ end were obtained from Qiagen. Cigarette smoke extract (CSE) was prepared by entrapping the smoke of two standard Research Cigarettes (The University of Kentucky Research Institute) in 25 ml of serum free media (DMEM/F12K) slowly over a period of 10 min. The CSE was filter sterilized and aliquot into 1 ml vials and frozen at −70°C, freshly thawed vials were used for each set of experiments.

### Cell culture

Marshall cell (MC), a fibroblastoid rat mammary tumor cell line (BICR-M1Rk) which expresses only Connexin43 (Cx43) was cultured in DMEM supplemented with 10% FCS and 1% penicillin-streptomycin as described [15]. L2 (Rat Lung Epithelial cell) and N2A (Mouse Neuroblastoma cell line) were purchased from ATCC, cultured and maintained as per their recommendations. Cells were split 36 hrs before the experiments on collagen coated chambered cover glass system (LabTek) at low density (2.0×10^3 ^cells/cm^2^) for hemichannel studies.

### Dye uptake studies

The presence of functional hemichannels was evaluated by LY uptake study as described [15]. Briefly, cells were incubated with 2 mM LY in the Ca^++^ free medium for 20 min at room temperature and washed with normal Ca^++^ [1.8 mM] containing medium, to close hemichannels and imaged. Dye uptake studies (LY) under oxidative stress conditions, induced by 10% CSE/1 mM H_2_O_2_, were performed in normal Ca^++^ containing medium with or without hemichannel blocker (βGA; 40 µM/MFA; 100 µM) or antioxidant (DTT; 10 mM). For experiments involving hemichannel blockers or antioxidants, cells were pretreated with these reagents for 20 min before carrying out dye uptake studies.

To rule out the possibility of dye uptake through non-hemichannel mediated pathways such as purinergic pathways or by membrane damage. LY uptake study was carried out in the presence of a broad spectrum purinergic blocker PPADS (10 µM) or O-ATP (300 µM) [50], and Texas Red conjugated Dextran (TR-Dx, 10 kDa; 2 mg/ml).

For quantitation, 10 randomly selected fields were chosen and the number of cells positive only for LY was counted and cells positive for both LY and TR-Dx were excluded from quantitation to control for LY uptake resulting from the loss of cell membrane integrity. The number of cells that uptake LY dye were counted and expressed as a percentage of the total number of cells counted. Dye uptake was examined by visual inspection using an inverted research microscope (Olympus, IX71) equipped with the appropriate filters under 20x magnification. All dye transfer assays were performed in triplicate. In Cx43-GFP transfected N2A cells, Ethidium Bromide (EtBr; 2.5 mM) was used instead of LY for hemichannel assay [18] in order to visualize Cx43-GFP and dye uptake simultaneously.

### Detection of cell membrane depolarization induced by oxidative stress

Membrane depolarization induced by oxidative stress (CSE/H_2_O_2_) was detected using the voltage sensitive dye DiBAC_4_(3). For this study, cells grown to near confluence on chambered cover glass were washed free of serum products with bicarbonate free HEPES buffer solution and loaded with 1 µM DiBAC_4_(3) for 30 min at room temperature. Cells were rinsed once to remove free unbound dye and its basal fluorescence was recorded. Subsequently, cells were challenged with 10% CSE/1 mM H_2_O_2 _with or without βGA/DTT and its fluorescence was measured at the end of 3 min (final fluorescence) from the time of application of CSE/H_2_O_2_. For experiments involving βGA and DTT, cells were pretreated with these reagents for 20 min prior to the application of CSE/H_2_O_2. _The fluorescence intensity value was derived from image segmentation analysis and the change in the fluorescence intensity was calculated by subtracting the basal fluorescence from final fluorescence and the data was plotted as percent increase in the basal fluorescence intensity against different treatments.

### Direct electrophysiological measurement of membrane potential

Marshall cells growing on glass bottomed Petri dishes (MatTek) were perfused by bicarbonate free HEPES buffer solution containing (in mM): 120 NaCl; 5 KCl; 2 CaCl_2_; 0.8 MgCl_2_; 0.9 Na_2_HPO_4_; 1 Sodium pyruvate; 10 D-Glucose; 20 HEPES; (pH 7.4). Membrane potentials were measured under current-clamp condition, using the whole-cell configuration with an Axopatch 200B amplifier (Axon Instruments) at room temperature. The patch pipettes had a resistance of 2-4 MΩ when filled with pipette solution containing (in mM): 110 KOH, 30 KCl, 5 NaCl, 10 HEPES, 1 EGTA, pH 7.2. After membrane potentials were measured under the control condition, the perfusion was switched to solutions with higher KCl concentrations (30, 60 and 120 mM, respectively). For dye uptake study, LY was added directly to the extracellular medium during perfusion and imaged. Data were filtered at 100 Hz and digitized at 200 Hz via a DigiData 1200 interface (Axon Instruments). Data acquisition and analysis were carried out using pCLAMP9 software (Axon Instruments).

### Live/Dead assay

Live/Dead assay was performed, as described [51], on MC treated with CSE, with and without hemichannel blockers (40 µM; βGA/100 µM; MFA) in OptiMEM I media using live/dead assay kit (Molecular probes). Cells were monitored hourly to assess their viability and the same fields were imaged until the end of the experiment. For quantitation, ten randomly selected fields were chosen and the number of live cells were counted and expressed as a percentage of the total number of cells counted.

### Intracellular oxidative stress detection using ROS sensitive dye

Intracellular oxidative stress due to the entry of ROS through hemichannels was detected by ROS sensitive dye (carboxy-H_2_DCFDA). Briefly, cells grown on collagen coated cover-slips were treated with H_2_O_2 _(1 mM) or CSE (10%) for 10 min at 37°C and washed to remove any free ROS from outside the cells and incubated with the ROS sensitive dye for 30 min at 37°C and imaged immediately. For βGA experiments, cells were pretreated for 20 min before applying H_2_O_2 _or CSE. The fluorescence intensity of different treatments was computed by image segmentation analysis. Replicate experiments were performed for each condition, and five randomly selected fields were chosen for analysis per condition, for a total of 10 measurements per condition. The mean fluorescence intensity was plotted and compared against the control.

### Cx43-GFP transfection in N2A cells

Cx43-GFP in pcDNA3.1 plasmid construct was transiently transfected into N2A cells using Lipofectamine 2000 as described in [15]. Briefly, 1 µg of DNA in 50 µl of Opti-MEM is mixed with 2 µl of Lipofectamine 2000 in 50 µl of OptiMEM and incubated for 20 min at room temperature. This mixture was added to the cells, grown on chambered cover glass system to near confluence, and further incubated for 36 hrs at 37°C in DMEM before carrying out experiments.

### siRNA transfection in Marshall Cell

siRNA transfection into Marshall cells was carried out as per the manufacturer's recommendations. Briefly, cells were cultured in 4 well chambers to 90% confluence. On the day of transfection, 1 µg of siRNA was mixed with HiPerFect transfection reagent and the complexes were incubated for 10 min at room temperature and added drop wise on the cells. The cells were further incubated for 24–36 hrs under normal growth conditions before using them for experiments. The number of apoptotic cells was expressed as a percentage of the total number of cells counted from 10 random fields. The data was further normalized for spot density to remove any discrepancies that may arise due to different densities employed in the transfected and untransfected groups by adjusting (upscale) the mean sample density of untransfected group to that of transfected group

### Data analysis

Images from the inverted microscope were captured by a high sensitivity, low noise and fast acquisition CCD camera (Qimaging, Retiga Exi, 12bit monochrome). All image analysis was carried out on the IPLab Image analysis software. Image deconvolution was performed with Huygens Essential deconvolution program (Scientific Volume Imaging). Statistical analyses were carried out in Excel and Analyze-it software packages. One way ANOVA with Dunnett's post hoc test was used to assess differences between treatment groups against their controls.

## Supporting Information

Figure S1 Detection of connexin hemichannels in non-junctional regions of the plasma membrane. The punctate fluorescence in the free margins of the cell membrane is very characteristic of connexin hemichannels. Cx43 was detected in MC (C), Cx32(F) and Cx43 (G) were detected in L2 cells, while no detectable connexins were found in N2A cells (I,J,K). No immunostaining was observed in cells incubated with secondary antibody alone without pre-incubation with primary antibody to serve as control (D,H,L). Scale bar: 5 µm
(0.05 MB PDF)

Figure S2 Dose dependent hemichannel opening induced by oxidative stress in Cx-expressing cells (MC). Dye uptake was seen for concentrations as low as 50 µM H^2^O^2^ (B) and 1% CSE (E). No significant LY uptake was seen at 25 µM H^2^O^2^ (C) and 0.5% CSE (F).
(0.07 MB PDF)

Figure S3 Double dye study to exclude the involvement of purinergic receptor pathways under oxidative stress. First, hemichannel opening induced by CSE was observed by EtBr uptake (B). In the second step, purinergic receptors were blocked with broad spectrum purinergic blocker (PPADS) and hemichannel opening was monitored by LY uptake under same stimuli (C), composite image shows both EtBr and LY positive cells (D) and phase image of the field is shown in (A). Scale bar: 20 µm.
(0.04 MB PDF)

Figure S4 Indirect exclusion of TRP channels in the dye transfer under oxidative stress. Cx-deficient N2A cells subjected to oxidative stress by CSE in the presence of broad spectrum purinergic blocker (PPADS) and the dye uptake was monitored by LY dye. To rule out non-specific dye uptake through compromised cell membranes, Texas-Red conjugated with Dextran (10 kDa) was included in the assay. No significant dye uptake was observed thus suggesting TRP channels are not involved in the dye uptake under oxidative stress. Scale bar: 20 µm.
(0.07 MB PDF)
