# Supplementary figures and images for: A Novel Role for Connexin Hemichannel in Oxidative Stress and Smoking-Induced Cell Injury
Source: PLoS One. 2007 Aug 8;2(8):e712. doi: 10.1371/journal.pone.0000712 (PMC1933596; doi:10.1371/journal.pone.0000712)

Figure S1

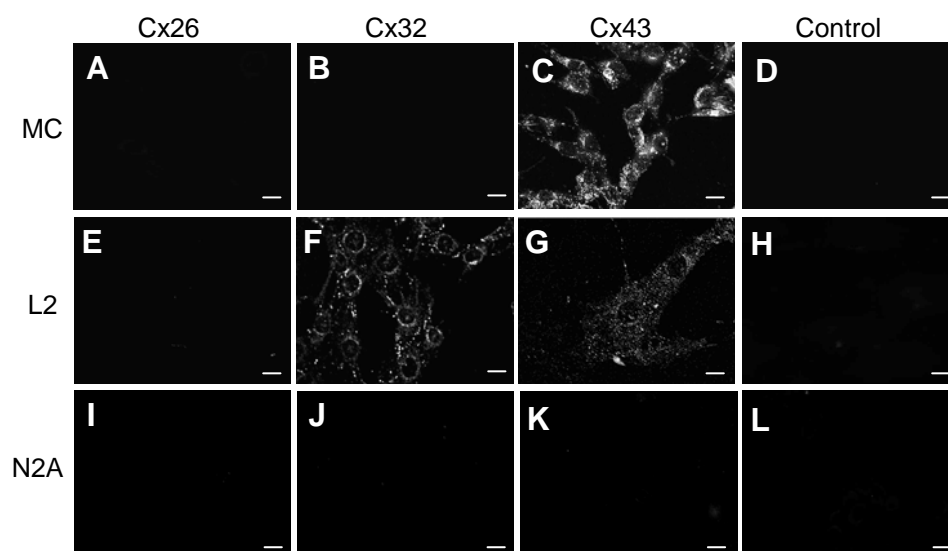

Supplement: Figure S1 — Detection of connexin hemichannels in non-junctional regions of the plasma membrane. The punctate fluorescence in the free margins of the cell membrane is very characteristic of connexin hemichannels. Cx43 was detected in MC (C), Cx32(F) and Cx43 (G) were detected in L2 cells, while no detectable connexins were found in N2A cells (I,J,K). No immunostaining was observed in cells incubated with secondary antibody alone without pre-incubation with primary antibody to serve as control (D,H,L). Scale bar: 5 µm (0.05 MB PDF) [file pone.0000712.s001.pdf]

Figure S2

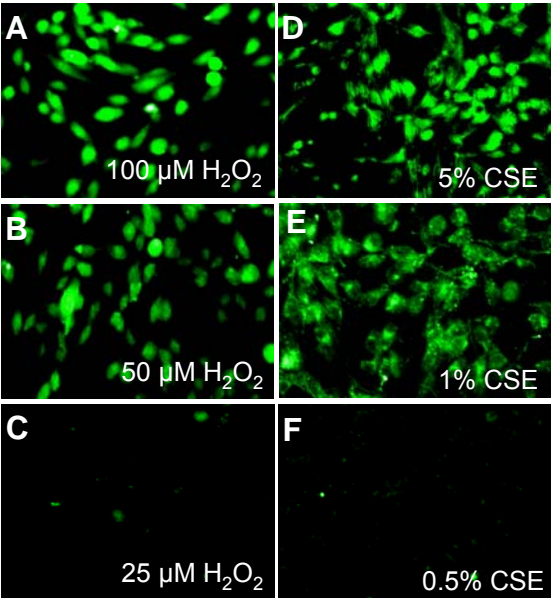

Supplement: Figure S2 — Dose dependent hemichannel opening induced by oxidative stress in Cx-expressing cells (MC). Dye uptake was seen for concentrations as low as 50 µM H2O2 (B) and 1% CSE (E). No significant LY uptake was seen at 25 µM H2O2 (C) and 0.5% CSE (F). (0.07 MB PDF) [file pone.0000712.s002.pdf]

Figure S3

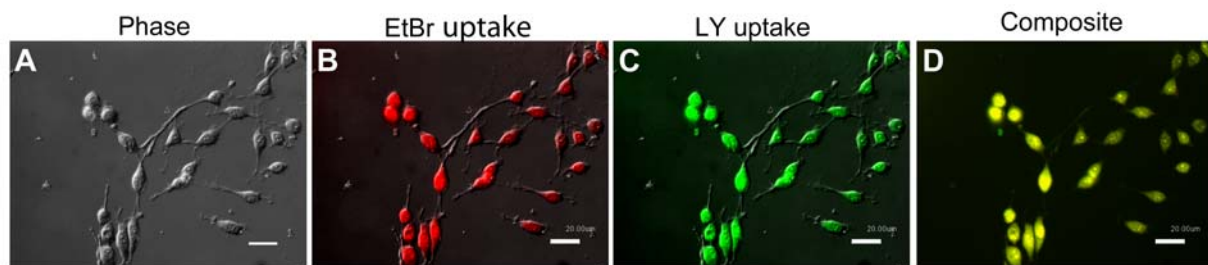

Supplement: Figure S3 — Double dye study to exclude the involvement of purinergic receptor pathways under oxidative stress. First, hemichannel opening induced by CSE was observed by EtBr uptake (B). In the second step, purinergic receptors were blocked with broad spectrum purinergic blocker (PPADS) and hemichannel opening was monitored by LY uptake under same stimuli (C), composite image shows both EtBr and LY positive cells (D) and phase image of the field is shown in (A). Scale bar: 20 µm. (0.04 MB PDF) [file pone.0000712.s003.pdf]

Figure S4

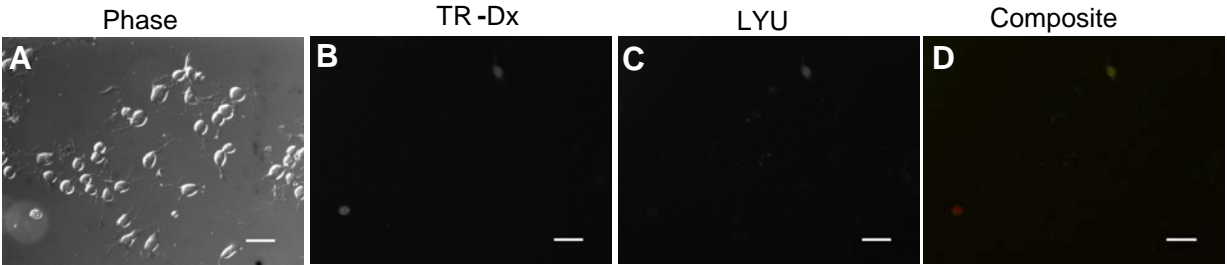

Supplement: Figure S4 — Indirect exclusion of TRP channels in the dye transfer under oxidative stress. Cx-deficient N2A cells subjected to oxidative stress by CSE in the presence of broad spectrum purinergic blocker (PPADS) and the dye uptake was monitored by LY dye. To rule out non-specific dye uptake through compromised cell membranes, Texas-Red conjugated with Dextran (10 kDa) was included in the assay. No significant dye uptake was observed thus suggesting TRP channels are not involved in the dye uptake under oxidative stress. Scale bar: 20 µm. (0.07 MB PDF) [file pone.0000712.s004.pdf]
